# Simplified Reversed Chloroquines To Overcome Malaria Resistance to Quinoline-Based Drugs

**DOI:** 10.1128/AAC.01913-16

**Published:** 2017-04-24

**Authors:** Bornface Gunsaru, Steven J. Burgess, Westin Morrill, Jane X. Kelly, Shawheen Shomloo, Martin J. Smilkstein, Katherine Liebman, David H. Peyton

**Affiliations:** a Department of Chemistry, Portland State University, Portland, Oregon, USA; b DesignMedix, Inc., Portland, Oregon, USA; c Portland Veterans Affairs Medical Center, Portland, Oregon, USA

**Keywords:** chloroquine, Plasmodium falciparum, antimalarial, drug discovery, accumulation, hemozoin, hematin, structure-activity relationship, drug development, malaria, drug resistance

## Abstract

Building on our earlier work of attaching a chemosensitizer (reversal agent) to a known drug pharmacophore, we have now expanded the structure-activity relationship study to include simplified versions of the chemosensitizer. The change from two aromatic rings in this head group to a single ring does not appear to detrimentally affect the antimalarial activity of the compounds. Data from *in vitro* heme binding and *β*-hematin inhibition assays suggest that the single aromatic RCQ compounds retain activities against Plasmodium falciparum similar to those of CQ, although other mechanisms of action may be relevant to their activities.

## INTRODUCTION

Malaria remains a major health problem, mainly in sub-Saharan Africa and parts of Asia and South America (1, 2), with over 200 million clinical infections and nearly half a million deaths annually (3). Malaria is caused by protozoan parasites belonging to the genus Plasmodium and is transmitted via the bite of a female Anopheles mosquito (4, 5). There are four major species of the parasite that cause malaria in humans, namely, Plasmodium falciparum, P. vivax, P. ovale, and P. malaria, while a fifth parasite, P. knowlesi, is now recognized (6, 7).

Historically, a range of drugs has been used to treat or prevent malaria (8–11), including several derived from the quinoline ring system. Examples include quinine, chloroquine (CQ), amodiaquine, piperaquine, mefloquine, and primaquine. All of these drugs have been suggested to act on the blood stages of the parasite's life cycle (12) except primaquine, an 8-aminoquinoline, which acts on the hepatic stage (13, 14).

CQ was introduced in the mid-20th century and quickly became the most important of the blood-stage-acting quinoline class of drugs. In addition to being generally safe, effective, and inexpensive, CQ could be used to treat children and pregnant women, who account for most of the deaths associated with malaria. However, resistance to CQ was reported as early as 1957 (15, 16), and today it is so widespread that CQ has been rendered almost ineffective as a therapy (17). CQ resistance is strongly correlated with mutations in the membrane protein P. falciparum chloroquine resistance transporter (PfCRT), located in the parasite's digestive vacuole (DV), the location of CQ's major antimalarial mode of action (18–21). There is evidence that CQ's accumulation in the DV is reduced in CQ-resistant (CQR) parasites, which has been implicated as a cause of the resistance (18, 19). PfCRT from CQR P. falciparum strains has been shown to be able to transport CQ, signifying a direct mechanistic link to CQR (22).

**FIG 1:**
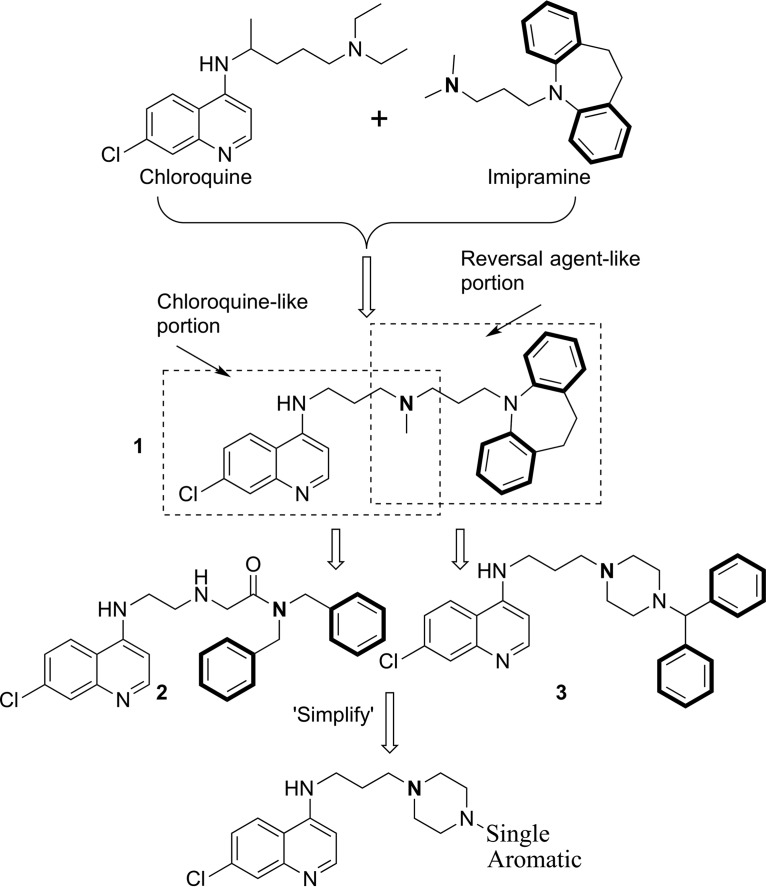
Evolution of the reversed chloroquine molecules. Highlighted in boldface are the two aromatic rings and the hydrogen bond acceptor of the reversal agent moiety. These are the key elements of the reversal agent pharmacophore, as identified by Bhattacharjee et al. (30).

However, verapamil, a calcium channel blocker, is able to block CQ transport by PfCRT (22) and has been shown to be able to reverse CQR in P. falciparum (23, 24). Certain tricyclic antidepressants (25), antihistamines (26), and some antiretroviral protease inhibitors (27–29) also possess this ability. A three-dimensional QSAR pharmacophore model for these chemosensitizers, or reversal agents (RAs), was developed, and it indicated that two aromatic hydrophobic interaction sites linked by an aliphatic chain to a hydrogen bond acceptor site (generally nitrogen) were required for activity (30). A hybrid “reversed-chloroquine” (RCQ) (Fig. 1) molecule was subsequently synthesized in our laboratory, consisting of a CQ-like moiety linked to an RA-like moiety (31). Due to the potency of this hybrid drug (50% inhibitory concentration [IC_50_] for CQ-sensitive [CQS] D6 and CQR Dd2 of 2.9 nM and 5.3 nM, respectively), work was started on the synthesis of related compounds in order to investigate the structure-activity relationship (SAR) (32, 33). Compounds 2 and 3 (Fig. 1) are two examples of the compounds generated during those studies; these compounds illustrate some of the changes to both the RA head group and the linker between the CQ-like moiety and the RA head group. Note that both examples retain the two aromatic rings in the RA head group, as suggested by the published pharmacophore (30). Others have recently reported efforts in developing similar CQ hybrid drug-like molecules (34).

There is a continuing need for the development of new but inexpensive malaria drugs with minimal toxicity or side effects, because the Plasmodium parasites continue to develop resistance to current chemotherapies, including even those of the artemisinin class (35, 36). The loss of the clinical usefulness of CQ, with its few side effects, high safety, high efficacy, and extremely low cost, is particularly regrettable.

Here, we report on RCQ-like molecules with a simplified head group moiety, having only a single aromatic ring, in order to investigate the possibility of lowering the cost of goods, simplifying syntheses, and/or increasing solubility. These new compounds therefore deviate from the published pharmacophore RA (30). However, and perhaps surprisingly, this set of simplified compounds generally has a marked improvement in potency that may lead to drugs with reduced dosages, lowered cost, and reduced toxicity.

## RESULTS

**FIG 2:**
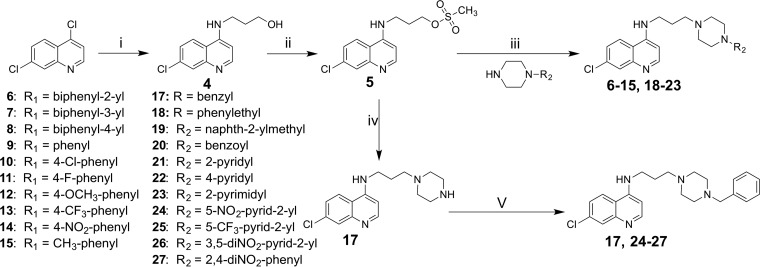
Synthetic approaches to the simplified reversed chloroquine molecules. Reagents and conditions included the following: (i) 3-aminopropanol, 130°C, 48 h; (ii) methanesulfonyl chloride, dichloromethane, Et_3_N, 0°C, 0.5 h; (iii) THF, Et_3_N, and reflux for 96 h, or acetonitrile, K_2_CO_3_, and reflux for 96 h; (iv) piperazine, THF, and reflux for 24 h; (v) halide compound, acetonitrile, K_2_CO_3_, and reflux.

The syntheses of compounds 4, 5, and 16 have been previously described (31, 33). Syntheses of other compounds are shown in Fig. 2. Compounds 6 to 15 and 18 to 23 were synthesized by treating compound 5 with the appropriate commercially available piperazine analogues. Compounds 17 and 24 to 27 were prepared by treating compound 16 with the appropriate halide in the presence of base.

**FIG 3:**
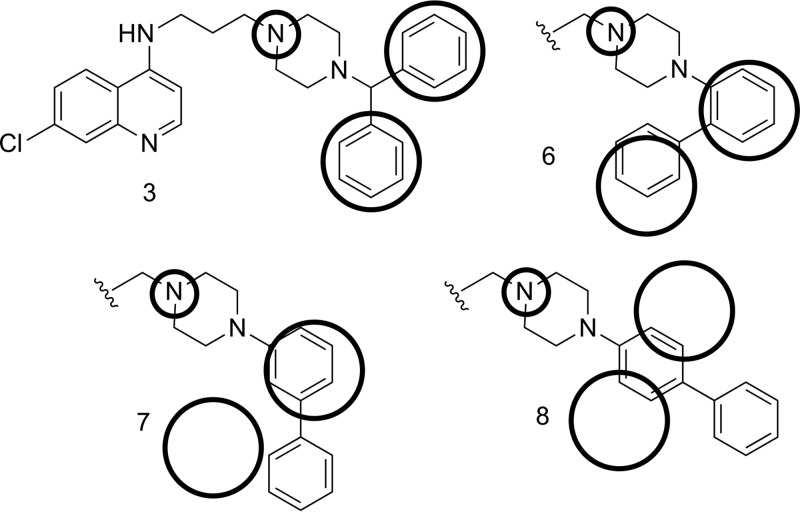
Changes in the orientation of the rings in the RA head group. The circles indicate how the orientation of the aromatic rings differs from the pharmacophore model.

**FIG 4:**
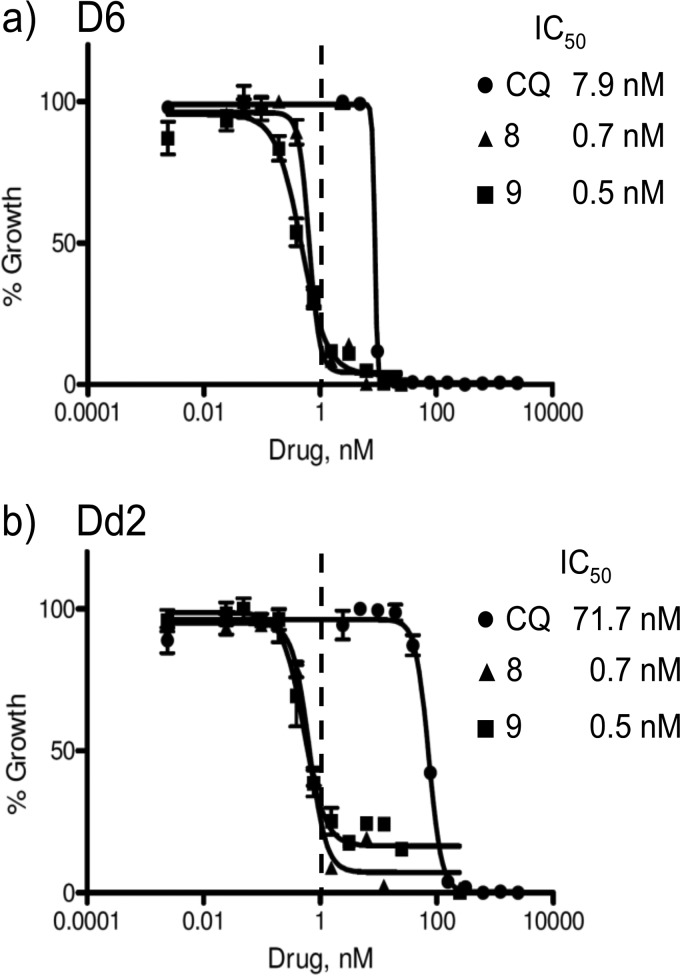
Dose-response curves for CQ and compounds 8 and 9 against CQS D6 (a) and CQR Dd2 (b). Parasite growth is measured in fluorescence units and normalized to the control values to give percent growth. The best-fit curves and IC_50_s were calculated by Prism (GraphPad) software.

Compounds 1 to 3 have good activity against both CQS and CQR P. falciparum, with the RA head group remaining faithful to our starting-point pharmacophore: a triangular orientation of the aromatic rings and nitrogen-hydrogen bond acceptor (e.g., compound 1, as illustrated in Fig. 3). We decided to investigate whether this orientation of the two RA aromatic rings was important for activity. Thus, compounds 6 to 8 were synthesized, having the orientation of the rings systematically changed until a linear arrangement was reached. The *in vitro* activities of these compounds were still good, and in the case of compound 8 they were surprisingly good, with low- to sub-nanomolar IC_50_s for each of the three strains tested (Table 1 and Fig. 4). As the linear orientation of the rings in compound 8 was quite different from the starting-point pharmacophore (Fig. 3), the decision was made to move even further away from that model and remove the second ring altogether, resulting in compound 9. This did not reduce the remarkably potent antimalarial activity (e.g., Fig. 4, IC_50_ data fits) but did reduce the calculated partition coefficient (ClogP) value to a number that is nearly that of CQ and therefore much more “drug-like” (Table 1).

**TABLE 1:**
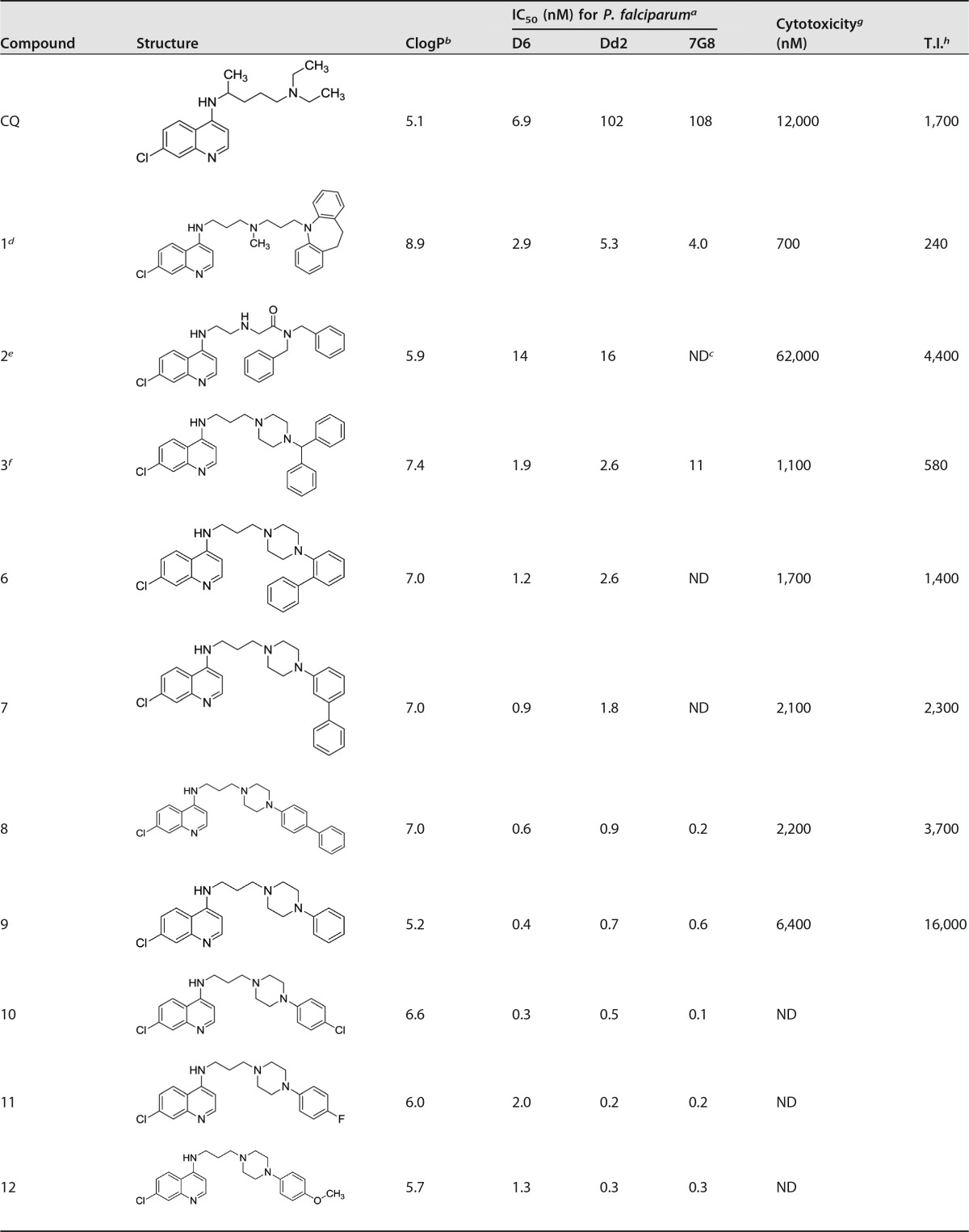

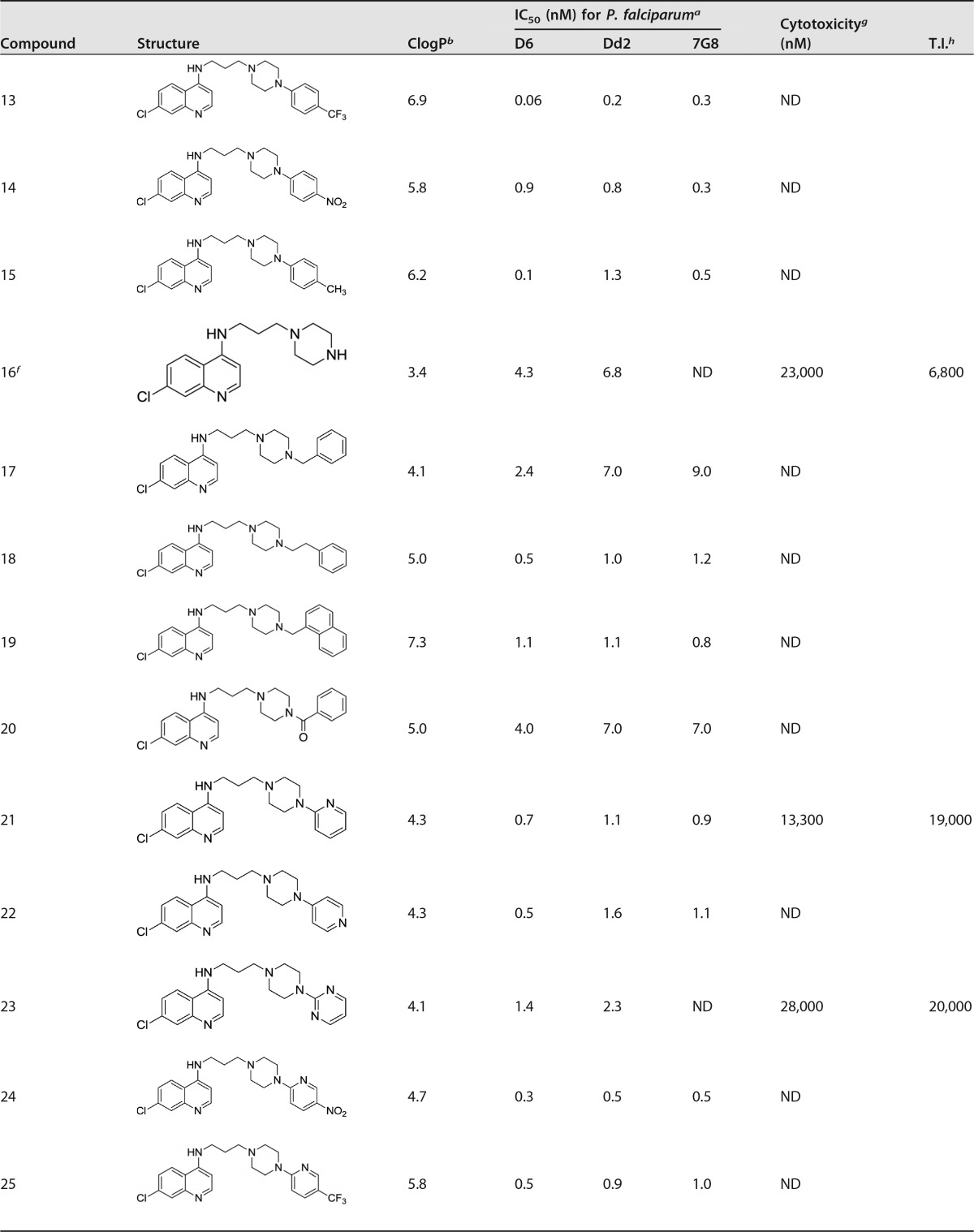

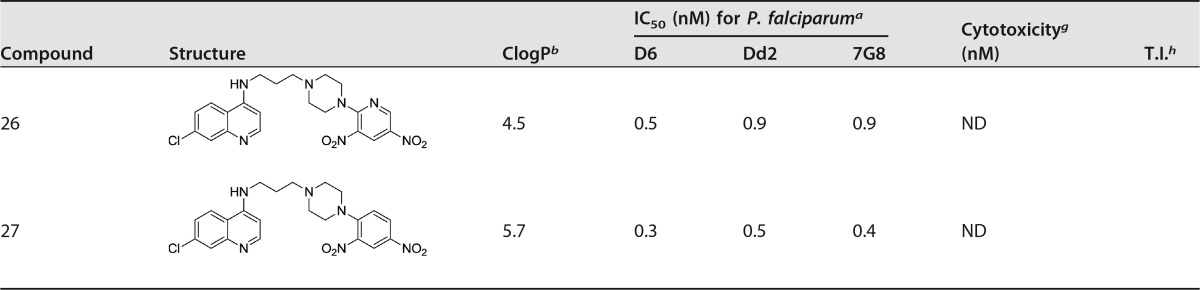
Structures, ClogP, IC_50,_ and cytotoxicity and therapeutic index values for CQ and synthesized compounds

^a^ IC_50_s shown are for CQS D6 and for CQR Dd2 and 7G8. Results are averages from at least 3 runs. The uncertainties were estimated to be ±15%, based on weighing uncertainties for the various compounds, as well as on variations between determinations that were performed during different weeks. In order to compare results run on different days and with different batches of each strain, CQ was run as a positive control; the results obtained were then normalized to the CQ values of 6.9 nM for D6, 102 nM for Dd2, and 108 nM for 7G8. For example, the normalized IC_50_ for an RCQ compound tested against a D6 strain was determined as [6.9/IC_50_ CQ (D6)] × IC_50_ of RCQ compound D6.

^b^ ClogP values were calculated using ChemDraw Ultra.

^c^ ND, not determined.

^d^ Burgess et al. (31).

^e^ Andrews et al. (32).

^f^ Burgess et al. (33).

^g^ Cytotoxicities are against mouse spleen lymphocytes. These values are estimated to be ±50%, based on weighing uncertainties for the various compounds (which are free bases and often oils), as well as on variability between determinations that were performed during different weeks.

^e^ T.I., therapeutic index (unitless; determined as cytotoxicity/efficacy against D6 of 12,000 for D6).

Given the surprisingly good activity of compound 9, a SAR study on the single aromatic RA version of the RCQ compounds was initiated. Compounds 10 to 15 were synthesized with various substituents on the single phenyl ring. While there was some variation in results, all had IC_50_s below 5 nM, and many were even below 1 nM (Table 1). Further variations on compound 9 led to the introduction of a second linker unit between the phenyl and piperazine rings (compounds 17, 18, and 20), as well as replacing the phenyl with naphthyl and heterocyclic ring systems, and including some substituted examples (compounds 24 to 27). None of these changes had any serious detrimental effects on *in vitro* activity, and several have ClogP values lowered to below 5. Of particular note are the pyridyl compounds 21 and 22 and the pyrimidine compound 23 (Table 1), all of which have the potential to form very water-soluble salts and may be especially orally available.

### *In vitro* heme binding and hemozoin inhibition.

We have previously shown that, in a simple *in vitro* test tube experiment, the RCQ compounds with the RA-like moiety containing two aromatic rings bind heme and inhibit *β*-hematin formation to an extent similar to that of CQ (33). Applying the same tests to a selection of these single aromatic RCQ compounds (compounds 9, 11, 12, and 13), we found that these compounds bind to heme to a similar extent as CQ, with all compounds having dissociation constant (*K_d_*) values of about 5 μM (CQ gives 5.4 μM in our test). In the *β*-hematin inhibition test, CQ had an IC_50_ of 35 μM, and the RCQ compounds all fell into the range of 5 to 43 μM (similar to CQ). These results point to the capability of these new RCQ compounds to act against P. falciparum in a manner similar to that of CQ.

### *In vivo* efficacy against Plasmodium yoelii.

A selection of the compounds (compounds 9, 10, 12, 21, 23, and 24) was tested *in vivo* against P. yoelii in a murine malaria model. The compounds were first converted to water-soluble hydrochloride or phosphate salts for administration in aqueous solution. Efficacy testing was carried out using 4 different dose levels with 5 mice at each level, administered by oral gavage. It can be seen from Table 2 that several of the compounds, such as compounds 9 and 21, have low 50% effective dose (ED_50_) values, indicating they are indeed orally efficacious. A single-dose toxicity evaluation was also performed, using 1 or 2 mice in each case. The doses were limited by water solubilities. Using this crude screen, the lack of evident toxicity at doses as high as 150-fold the ED_50_ (compound 9) suggests a favorable safety margin.

**TABLE 2:**
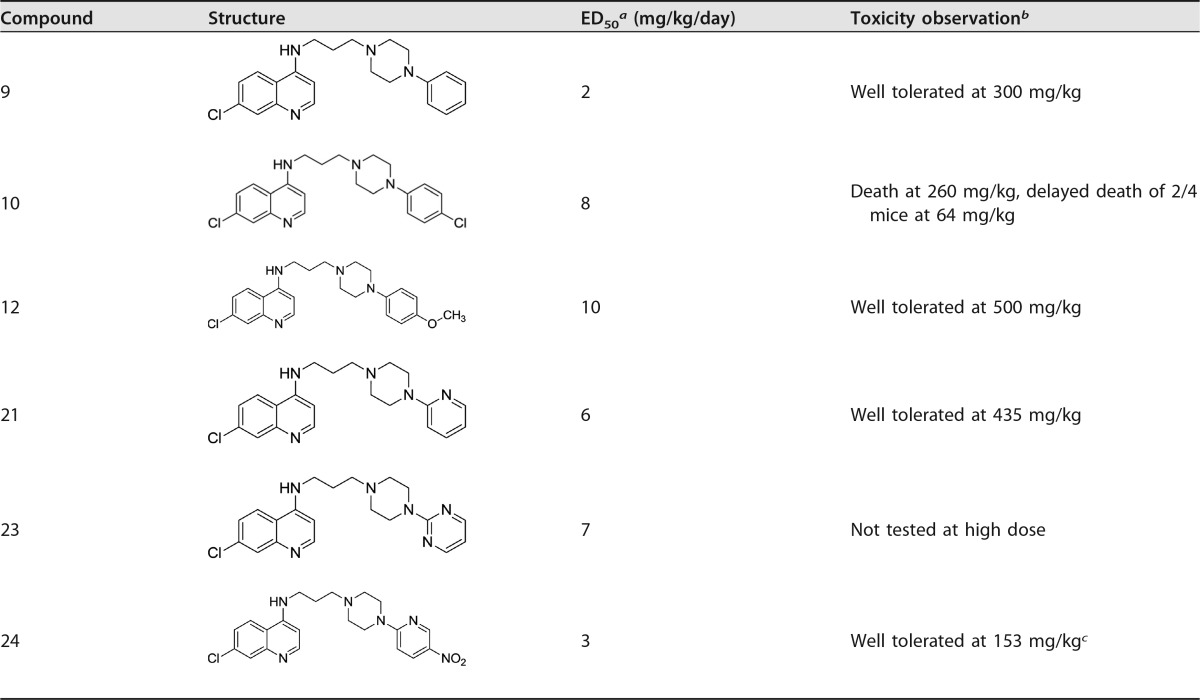
*In vivo* 3-day suppressive test against P. yoelii in a murine malaria model

^a^ Experiments consisted of at least 4 doses, with 4 or 5 mice/dose. ED_50_s were calculated by Prism (GraphPad) software from a best-fit curve. Errors are estimated to be ±20% and reported to the nearest integer value.

^b^ Toxicity observations were obtained from single-dose experiments on 1 or 2 mice/compound.

^c^ This was the highest dose possible given the reduced aqueous solubility of this compound.

## DISCUSSION

The change from two aromatic rings in the RA-like head group to a single ring does not appear to have detrimentally affected the antimalarial activity of the RCQ compounds, perhaps to a surprising extent. The data from the *in vitro* heme binding and *β*-hematin inhibition tests suggest that the single aromatic RCQ compounds can still act against P. falciparum in a manner similar to that of CQ, although further mechanistic testing in the parasite is required to confirm whether they actually do so (the *in vitro* results cannot provide proof of this). As is the case for many drugs, whether other mechanisms of action also are relevant to their activities remains an open question. Nevertheless, *in vivo* results are very encouraging at this early stage. Several of the compounds were evaluated for their cytotoxicity potential in a mammalian system (Table 1). Examining the data in Table 1, it becomes apparent that there is a correlation between ClogP and cytotoxicity. In fact, the compounds with the highest therapeutic index (cytotoxicity/antimalarial potency ratio) are generally those compounds with the lowest ClogP values (and, thus, the least lipophilic compounds). It is notable that many of the most potent of the compounds (lowest IC_50_s) are the most lipophilic but also are the most cytotoxic. Thus, the choice for moving candidates forward in a drug development pathway would necessarily be a compromise between such competing factors. In fact, *in vivo* evaluations of potencies and toxicities may be more important than these *in vitro* screening tests to the selection of compounds for further development.

There are several compounds shown in Table 2 that demonstrate good oral efficacy (and potency) without evident toxicity when administered as salts in aqueous solution. Of course, there remain many more preclinical evaluations in order to choose and validate lead drug candidates. What this study provides is a group of very synthetically accessible and inexpensive compounds that overcome drug resistance of well-accepted test strains, as well as low cytotoxicity and little acute toxicity in a single mammalian system. An encouraging step in this evaluation process is our recent report that one of these simplified compounds, as well as a compound containing two aromatic rings, both have good potency against *ex vivo* clinical isolates infected by either P. falciparum or P. vivax malaria parasites (37).

## MATERIALS AND METHODS

All chemicals were obtained from Sigma-Aldrich Chemical Co. or TCI America and were used as supplied. Purities of all final products were ≥95% as determined by high-performance liquid chromatography (HPLC), measured by UV detection at 254 and 325 nm with a Varian ProStar 325 UV-visible dual-wavelength detector. HPLC was done with a Microsorb-MV 100-5 C_18_ 250-mm by 4.6-mm column. Elution was done with 95% methanol and 5% water with 0.1% TFA for 30 min. HPLC method C was performed using a Supelco Ascentis C_18_ column (5-μm volume; 4.6 mm by 150 mm), eluting with a 30-min gradient from 95:5 to 5:95 water with 0.1% (vol/vol) formic acid–acetonitrile. Retention times (*t*_R_) are given in minutes. High-resolution mass spectrometry was performed on a Bruker micrOTOF-Q instrument. Results were obtained using electrospray ionization mass spectrometry (ESIMS) in the positive mode at a flow rate of 0.4 ml/min with 1:1 methanol-water. ^1^H, ^13^C, and two-dimensional nuclear magnetic resonance (NMR) experiments were run on a Bruker 400 MHz Avance II+ instrument using the standard pulse sequences provided, including zg30, zgpg30 cosygpqf, hsqcetgpsi2, hmbcgplpndqf, and noesyph, at 25°C.

The syntheses of compounds 4, 5, and 16 have been previously described (31, 33).

### General procedure A for the preparation of compounds 6 to 9, 11, 15, 18, 19, 22, and 23.

Compound 5 was added to a mixture of the respective piperazine and triethylamine (Et_3_N) in tetrahydrofuran (15 ml). The reaction mixture was allowed to reflux for 4 days, cooled, and then poured into saturated sodium bicarbonate solution (30 ml). The resulting mixture was extracted with chloroform (3 times with 10 ml each time). The combined chloroform extracts were evaporated, and the crude product was purified by recrystallization and/or chromatography.

### General procedure B for the preparation of compounds 10, 12 to 14, and 21.

Compound 5 was added to a mixture of the respective piperazine and K_2_CO_3_ in acetonitrile, and the reaction mixture was heated to reflux for 72 h. After cooling to room temperature, the solvent was removed and the residue partitioned between water and chloroform. The organic layer was separated, washed with water, and then dried and evaporated to leave the crude product, which was then purified by recrystallization and/or chromatography.

### *N*-(3-(4-(Biphenyl-2-yl)piperazin-1-yl)propyl)-7-chloroquinolin-4-amine (compound 6).

The title compound was prepared from compound 5 (0.60 g, 1.9 mmol), 1-(biphenyl-2-yl)piperazine (0.55 g, 2.29 mmol), and triethylamine (0.39 g, 3.81 mmol) according to general procedure A. The crude product was recrystallized from methanol-ethyl acetate (25:75) to give a brown powder (0.12 g, 14%). HPLC *t*_R_ = 11.38 (95% pure). ^1^H NMR δ (ppm) (DMSO-d6): 8.38 (1H, d, J = 5.37 Hz), 8.22 (1H, d, J = 9.04 Hz), 7.77 (1H, d, J = 2.20 Hz), 7.59 to 7.62 (2H, m), 7.40 to 7.42 (3H, m), 7.24 to 7.37 (3H, m), 7.16 to 7.21 (1H, m), 7.06 to 7.08 (2H, m), 6.46 (1H, d, J = 5.45 Hz), 3.27 to 3.29 (2H, m), 2.67 to 2.89 (4H, m), 2.37 (2H, m), 2.24 to 2.38 (4H, m), 1.76 to 1.78 (2H, m). ^13^C NMR δ (ppm) (CHCl_3_-d): 151.7, 150.7, 149.9, 141.0, 135.2, 134.9, 131.7, 128.9, 128.6, 128.4, 128.2, 126.8, 124.9, 123.1, 122.3, 118.0, 117.4, 98.4, 58.7, 53.7, 51.1, 44.4, 23.4. ESIMS [M + H]^+^ calculated for C_28_H_29_ClN_4_ 457.2151, found 457.2147.

### *N*-(3-(4-(Biphenyl-3-yl)piperazin-1-yl)propyl)-7-chloroquinolin-4-amine (compound 7).

The title compound was prepared from compound 5 (0.60 g, 1.9 mmol), 1-(biphenyl-3-yl)piperazine (0.39 g, 3.81 mmol), and triethylamine (0.39 g, 3.81 mmol) according to general procedure A. The crude product was recrystallized from methanol-ethyl acetate (25:75) to give a white solid (0.35 g, 18.4%). HPLC *t*_R_ = 11.51 (98% pure). ^1^H NMR δ (ppm) (CHCl_3_-d): 8.52 (1H, d, J = 5.35 Hz), 7.93 (1H, d, J = 2.15 Hz), 7.82 (1H, d, J = 8.94 Hz), 7.63 to 7.58 (2H, m), 7.47 to 7.32 (5H, m), 7.24 (1H, dd, J = 8.00, 2.28 Hz), 7.20 to 7.13 (2H, m), 6.98 (1H, dd, J = 8.30, 2.49 Hz), 6.36 (1H, d, J = 5.41 Hz), 3.43 (2H, d, J = 5.08 Hz), 3.42 to 3.37 (4H, m), 2.77 (4H, m), 2.74 to 2.68 (2H, m), 2.05 to 1.97 (2H, m). ^13^C NMR δ (ppm) (CHCl_3_-d): 160.9, 152.4, 151.6, 147.9, 145.3, 141.4, 134.6, 129.8, 129.2, 127.5, 125.3, 121.9, 122.3, 119.6, 115.4, 113.7, 112.5, 98.4, 96.4, 58.8, 53.7, 49.5, 44.1, 24. ESIMS [M + H]^+^ calculated for C_28_H_29_ClN_4_ 457.2154, found 457.2171.

### *N*-(3-(4-(Biphenyl-4-yl)piperazin-1-yl)propyl)-7-chloroquinolin-4-amine (compound 8).

The title compound was prepared from compound 5 (0.50 g, 1.59 mmol), 1-(biphenyl-4-yl)piperazine (0.45 g, 1.91 mmol), and triethylamine (0.32 g, 3.17 mmol) according to general procedure A. The crude product was purified by column chromatography on silica, eluting with methanol-ethyl acetate (1:3), to give a white solid (0.16 g, 22%). HPLC *t*_R_ = 10.84 (96% pure). ^1^H NMR δ (ppm) (DMSO-d6): 8.41 (1H, d, J = 5.46 Hz), 8.27 (1H, d, J = 9.02 Hz), 7.79 (1H, d, J = 2.25 Hz), 7.58 to 7.62 (2H, m), 7.53 to 7.55 (2H, m), 7.48 (1H, br t, J = 5.76 Hz), 7.44 (1H, dd, J = 9.01, 2.34 Hz), 7.40 to 7.42 (2H, m), 7.26 to 7.27 (1H, m), 6.99 to 7.04 (2H, m), 6.53 (1H, d, J = 5.53 Hz), 3.33 to 3.38 (~2H, m), 3.18 to 3.25 (4H, m), 2.55 to 2.59 (4H, m), 2.46 to 2.50 (2H, m), 1.85 to 1.90 (2H, m). ^13^C NMR δ (ppm) (DMSO-d6): 152.1, 150.8, 140.5, 134.0, 130.8, 129.3, 127.6, 127.6, 126.8, 126.3, 124.6, 117.9, 116.0, 99.1, 56.0, 53.2, 48.5, 41.3, 25.5. ESIMS [M + H]^+^ calculated for C_28_H_29_ClN_4_ 457.2154, found 457.2143.

### 7-Chloro-*N*-(3-(4-phenylpiperazin-1-yl)propyl)quinolin-4-amine (compound 9).

The title compound was prepared from compound 5 (0.30 g, 1.0 mmol), 1-phenylpiperazine (0.32 g, 1.2 mmol), and triethylamine (0.27 g, 2.0 mmol) according to general procedure A. The crude product was recrystallized from ethanol to give a cream solid (0.16 g, 44%). HPLC *t*_R_ = 8.28 (99% pure). ^1^H NMR δ (ppm) (CHCl_3_-d): 8.51 (1H, d, J = 5.35 Hz), 7.92 (1H, d, J = 2.16 Hz), 7.80 (1H, d, J = 8.92 Hz), 7.33 to 7.34 (3H, m), 7.20 (1H, dd, J = 8.89, 2.18 Hz), 6.96 to 7.01 (2H, m), 6.90 to 6.95 (1H, m), 6.34 (1H, d, J = 5.39 Hz), 3.41 (2H, td, J = 5.93, 4.21 Hz), 3.32 to 3.33 (4H, m), 2.73 to 2.76 (4H, m), 2.69 (2H, t, J = 5.41 Hz), 2.00 (2H, m). ^13^C NMR δ (ppm) (CHCl_3_-d): 152.2, 151.1, 150.5, 149.1, 134.7, 129.3, 128.7, 124.8, 122.0, 120.3, 117.4, 116.2, 98.6, 58.6, 53.7, 49.4, 44.3, 23.6. ESIMS [M + H]^+^ calculated for C_22_H_25_ClN_4_ 381.1841, found 381.1831.

### 7-Chloro-*N*-(3-(4-(4-chlorophenyl)piperazin-1-yl)propyl)quinolin-4-amine (compound 10).

The title compound was prepared from compound 5 (15.0 g, 47.6 mmol), 1-(4-chlorophenyl)piperazine hydrochloride (12.22 g, 52.4 mmol), and K_2_CO_3_ (14.5 g, 104.8 mmol) in acetonitrile (150 ml) according to general procedure B. The crude product was recrystallized from ethanol to give a solid (14 g, 71%). HPLC *t*_R_ = 5.91 (98% pure). ^1^H NMR δ (ppm) (CHCl_3_-d): 8.52 (1H, d, J = 5.34 Hz), 7.93 (1H, d, J = 2.14 Hz), 7.77 (1H, d, J = 8.92 Hz), 7.29 to 7.27 (~2H, m), 7.24 (1H, br t, J = 4.06 Hz), 7.20 (1H, dd, J = 8.89, 2.16 Hz), 6.92 to 6.87 (2H, m), 6.35 (1H, d, J = 5.38 Hz), 3.41 (2H, td, J = 5.93, 4.28 Hz), 3.27 to 3.30 (4H, m), 2.72 to 2.73 (4H, m), 2.69 (2H, t, J = 5.43 Hz), 2.00 (2H, m). ^13^C NMR δ (ppm) (CHCl_3_-d): 152.2, 150.4, 149.7, 149.2, 134.7, 129.2, 128.8, 125.1, 124.8, 121.9, 117.4, 117.3, 98.6, 58.5, 53.5, 49.4, 44.2, 23.7. ESIMS [M +H]^+^ calculated for C_22_H_24_Cl_2_N_4_ 415.1451, found 415.1460.

### 7-Chloro-*N*-(3-(4-(4-fluorophenyl)piperazin-1-yl)propyl)quinolin-4-amine (compound 11).

The title compound was prepared from compound 5 (0.58 g, 1.84 mmol), 1-(4-fluorophenyl)piperazine (0.40 g, 2.21 mmol), and triethylamine (0.37 g, 3.68 mmol) according to general procedure A. The crude product was recrystallized from methanol-ethyl acetate (25:75) to give a white powder (0.26 g, 35%). HPLC *t*_R_ = 5.91 (99% pure). ^1^H NMR δ (ppm) (CHCl_3_-d): 8.52 (1H, d, J = 5.36 Hz), 7.93 (1H, d, J = 2.16 Hz), 7.81 (1H, d, J = 8.94 Hz), 7.31 (1H, br t, J = 4.04 Hz), 7.21 (1H, dd, J = 8.91, 2.17 Hz), 7.02 to 7.03 (2H, m), 6.94 to 6.94 (2H, m), 6.35 (1H, d, J = 5.40 Hz), 3.42 (2H, td, J = 5.91, 4.26 Hz), 3.25 to 3.26 (4H, m), 2.72 to 2.77 (4H, m), 2.69 to 2.70 (2H, m), 2.00 (2H, m). ESIMS [M +H]^+^ calculated for C_22_H_24_ClFN_4_ 399.1746, found 399.1753.

### 7-Chloro-*N*-(3-(4-(4-methoxyphenyl)piperazin-1-yl)propyl)quinolin-4-amine (compound 12).

The title compound was prepared from compound 5 (10.00 g, 31.7 mmol), 1-(4-methoxyphenyl)piperazine dihydrochloride (9.27 g, 35.0 mmol), and K_2_CO_3_ (13.17 g, 95.3 mmol) in acetonitrile (100 ml) according to general procedure B. The crude product was recrystallized from ethanol to give a solid (7.2 g, 55%). HPLC *t*_R_ = 6.88 (98% pure). ^1^H NMR δ (ppm) (CHCl_3_-d): 8.51 (1H, d, J = 5.35 Hz), 7.92 (1H, d, J = 2.14 Hz), 7.83 (1H, d, J = 8.92 Hz), 7.43 (1H, br t, J = 4.06 Hz), 7.21 (1H, dd, J = 8.88, 2.16 Hz), 6.94 to 6.98 (2H, m), 6.89 to 6.91 (2H, m), 6.34 (1H, d, J = 5.39 Hz), 3.81 (3H, s), 3.41 (2H, td, J = 5.87, 4.17 Hz), 3.22 to 3.23 (4H, m), 2.73 to 2.76 (4H, m), 2.70 (2H, t, J = 5.32 Hz), 1.99 (2H, m). ^13^C NMR δ (ppm) (CHCl_3_-d): 154.2, 152.2, 150.5, 149.2, 145.5, 134.7, 128.7, 124.8, 122.2, 118.3, 117.5, 114.6, 98.6, 58.7, 55.6, 53.8, 50.9, 44.4, 23.6. ESIMS [M + H]^+^ calculated for C_23_H_27_ClN_4_O 411.1946, found 411.1949.

### 7-Chloro-*N*-(3-(4-(4-(trifluoromethyl)phenyl)piperazin-1-yl)propyl)quinolin-4-amine (compound 13).

The title compound was prepared from compound 5 (2.03 g, 6.44 mmol), 1-(4-trifluoromethylphenyl)piperazine (1.63 g, 7.08 mmol), and K_2_CO_3_ (1.07 g, 7.73 mmol) in acetonitrile (30 ml) according to general procedure B. The crude product was recrystallized from ethanol to give a solid (0.36 g, 13%). HPLC *t*_R_ = 5.88 (99% pure). ^1^H NMR δ (ppm) (CHCl_3_-d): 8.52 (1H, d, *J* = 5.35 Hz), 7.93 (1H, d, *J* = 2.16 Hz), 7.75 (1H, d, *J* = 8.94 Hz), 7.54 (2H, d, *J* = 8.60 Hz), 7.20 (1H, dd, *J* = 8.90, 2.17 Hz), 7.11 (1H, s), 6.98 (2H, d, *J* = 8.59 Hz), 6.36 (1H, d, *J* = 5.39 Hz), 3.46 to 3.38 (6H, m), 2.74 (4H, t, *J* = 4.91 Hz), 2.72 to 2.66 (2H, m), 2.01 (2H, p, *J* = 5.65 Hz). ESIMS [M + H]^+^ calculated for C_23_H_24_ClF_3_N_4_ 449.1714, found 449.1729.

### 7-Chloro-*N*-(3-(4-(4-nitrophenyl)piperazin-1-yl)propyl)quinolin-4-amine (compound 14).

The title compound was prepared from compound 5 (4.45 g, 14.15 mmol), 1-(4-nitrophenyl)piperazine (3.22 g, 15.57 mmol), and K_2_CO_3_ (2.15 g, 15.57 mmol) in acetonitrile (60 ml) according to general procedure B. The crude product was recrystallized from ethanol to give a solid (5.1 g, 68%). HPLC *t*_R_ = 8.93 (95% pure). ^1^H NMR δ (ppm) (CHCl_3_-d): 8.53 (1H, d, J = 5.36 Hz), 8.14 to 8.19 (2H, m), 7.95 (1H, d, J = 2.14 Hz), 7.72 (1H, d, J = 8.92 Hz), 7.23 (1H, dd, J = 8.88, 2.14 Hz), 6.87 to 6.89 (2H, m), 6.85 (1H, br t, J = 4.14 Hz), 6.39 (1H, d, J = 5.39 Hz), 3.52 to 3.53 (4H, m), 3.44 (2H, td, J = 6.07, 4.46 Hz), 2.70 to 2.73 (4H, m), 2.69 (2H, t, J = 5.66 Hz), 2.01 (2H, m). ^13^C NMR δ (ppm) (CHCl_3_-d): 159.7, 154.9, 141.4, 139.4, 132.7, 130.9, 129.3, 122.7, 108.4, 103.8, 96.7, 60.8, 58.6, 57.8, 51.6, 46.7, 4.8. ESIMS [M + H]^+^ calculated for C_22_H_24_Cl_1_N_5_O_2_ 426.1691, found 426.1682.

### 7-Chloro-*N*-(3-(4-*p*-tolylpiperazin-1-yl)propyl)quinolin-4-amine (compound 15).

The title compound was prepared from compound 5 (0.70 g, 2.22 mmol), 1-(4-methylphenyl)piperazine (0.47 g, 2.67 mmol), and triethylamine (0.45 g, 4.44 mmol) according to general procedure A. The crude product was recrystallized from methanol-ethyl acetate (25:75) to give yellow crystals (0.1 g, 12%). HPLC *t*_R_ = 6.67 (∼100% pure). ^1^H NMR δ (ppm) (CHCl_3_-d): 8.51 (1H, d, J = 5.35 Hz), 7.92 (1H, d, J = 2.15 Hz), 7.82 (1H, d, J = 8.92 Hz), 7.41 (1H, br t, J = 4.15 Hz), 7.21 (1H, dd, J = 8.88, 2.17 Hz), 7.09 to 7.18 (2H, m), 6.87 to 6.92 (2H, m), 6.34 (1H, d, J = 5.38 Hz), 3.41 (2H, td, J = 5.89, 4.16 Hz), 3.26 to 3.29 (4H, m), 2.72 to 2.75 (4H, m), 2.69 (2H, t, J = 5.33 Hz), 2.31 (3H, s), 1.98 to 1.99 (2H, m). ^13^C NMR δ (ppm) (DMSO-d6): 152.4, 150.6, 149.6, 149.5, 133.8, 129.8, 128.0, 128.0, 124.5, 124.4, 117.9, 116.1, 99.1, 56.1, 53.4, 49.2, 41.3, 25.5, 20.5. ESIMS [M + H]^+^ calculated for C_23_H_27_ClN_4_ 395.1997, found 395.1984.

### 7-Chloro-*N*-(3-(4-phenethylpiperazin-1-yl)propyl)quinolin-4-amine (compound 18).

The title compound was prepared from compound 5 (0.60 g, 1.91 mmol), 1-phenethylpiperazine (0.44 g, 2.28 mmol), and triethylamine (0.38 g, 3.81 mmol) according to general procedure A. The crude product was recrystallized from ethyl acetate to give a tan solid (0.29 g, 37%). HPLC *t*_R_ = 9.43 (95% pure). ^1^H NMR δ (ppm) (CHCl_3_-d): 8.51 (1H, d, J = 5.35 Hz), 7.94 (1H, d, J = 2.14 Hz), 7.88 (1H, d, J = 8.91 Hz), 7.55 (1H, br t, J = 4.04 Hz), 7.29 to 7.36 (3H, m), 7.18 to 7.29 (~3H, m), 6.33 (1H, d, J = 5.39 Hz), 3.39 (2H, td, J = 5.82, 4.08 Hz), 2.74 to 2.75 (14H, m), 1.93 to 1.98 (2H, m). ^13^C NMR δ (ppm) (CHCl_3_-d): 152.2, 150.6, 149.2, 140.1, 134.7, 128.7, 128.7, 128.5, 126.2, 124.7, 122.4, 117.5, 98.5, 60.7, 58.8, 53.6, 53.4, 44.5, 33.7, 23.4. ESIMS [M + H]^+^ calculated for C_23_H_29_ClN_4_ 409.2154, found 409.2156.

### 7-Chloro-*N*-(3-(4-(naphthalen-1-ylmethyl)piperazin-1-yl)propyl)quinolin-4-amine (compound 19).

The title compound was prepared from compound 5 (0.70 g, 2.22 mmol), 1-(naphthalene-1-ylmethyl)piperazine (0.60 g, 2.67 mmol), and triethylamine (0.45 g, 4.44 mmol) according to general procedure A. The crude product was recrystallized from ethyl acetate-methanol (3:1) to give yellow crystals (0.12 g, 12%). HPLC *t*_R_ = 7.21 (99% pure). ^1^H NMR δ (ppm) (CHCl_3_-d): 8.51 (1H, d, J = 5.31 Hz), 8.33 (1H, d, J = 8.36 Hz), 7.96 (1H, d, J = 2.14 Hz), 7.93 (1H, d, J = 8.93 Hz), 7.87 (1H, d, J = 8.06 Hz), 7.81 (1H, d, J = 8.10 Hz), 7.63 (1H, br t, J = 4.03 Hz), 7.52 to 7.52 (3H, m), 7.43 to 7.45 (1H, m), 7.35 (1H, dd, J = 8.90, 2.17 Hz), 6.32 (1H, d, J = 5.35 Hz), 4.03 (2H, s), 3.37 (2H, td, J = 5.78, 4.05 Hz), 2.63 to 2.64 (10H, m), 1.93 (2H, m). ^13^C NMR δ (ppm) (CHCl_3_-d): 23.4, 44.6, 53.3, 53.7, 58.8, 61.4, 98.5, 117.5, 122.6, 124.6, 124.7, 125.2, 125.7, 125.8, 127.6, 128.2, 128.5, 128.7, 132.6, 133.6, 133.9, 134.6, 149.2, 150.6, 152.3. ESIMS [M + H]^+^ calculated for C_27_H_29_ClN_4_ 445.2154, found 445.2154.

### *N*-(3-(4-Benzoylpiperazin-1-yl)propyl)-7-chloroquinolin-4-amine (compound 20).

The title compound was prepared from compound 5 (1.5 g, 4.8 mmol), benzoylpiperazine (0.95 g, 5.0 mmol), and K_2_CO_3_ (0.73 g, 5.25 mmol) in acetonitrile (25 ml), according to general procedure B. The crude product was purified by column chromatography on alumina, eluting with ethyl acetate, to give a solid (0.6 g, 30%). HPLC *t*_R_ = 6.81 (99% pure). ^1^H NMR δ (ppm) (CHCl_3_-d): 8.53 (1H, d, J = 5.36 Hz), 7.96 (1H, d, J = 2.15 Hz), 7.73 (1H, d, J = 8.94 Hz), 7.42-7.43 (5H, m), 7.38 (1H, dd, J = 8.91, 2.17 Hz), 6.74 (1H, br t, J = 4.39 Hz), 6.38 (1H, d, J = 5.40 Hz), 3.88 (2H, br m), 3.58 (2H, br m), 3.41 (2H, td, J = 6.11, 4.50 Hz), 2.63 to 2.65 (6H, m), 1.97 (2H, m). ^13^C NMR δ (ppm) (CHCl_3_-d): 170.4, 152.2, 150.2, 149.2, 135.5, 134.8, 129.9, 128.9, 128.6, 127.1, 125.1, 121.3, 117.4, 98.8, 58.1, 43.7, 24.0. ESIMS [M + H]^+^ calculated for C_23_H_25_ClN_4_O, 409.17897, found 409.17826.

### 7-Chloro-*N*-(3-(4-(pyridin-2-yl)piperazin-1-yl)propyl)quinolin-4-amine (compound 21).

The title compound was prepared from compound 5 (14.80 g, 47.0 mmol), 1-(2-pyridyl)piperazine (8.44 g, 51.7 mmol), and K_2_CO_3_ (7.15 g, 51.7 mmol) in acetonitrile (150 ml) according to general procedure B. The crude product was recrystallized from ethanol to give a solid (5.1 g, 68%). HPLC *t*_R_ = 5.49 (96% pure). ^1^H NMR δ (ppm) (CH_3_OH-d4): 8.39 (1H, d, J = 5.64 Hz), 8.10 to 8.11 (2H, m), 7.80 (1H, d, J = 2.18 Hz), 7.59 (1H, ddd, J = 8.64, 7.13, 2.00 Hz), 7.40 (1H, dd, J = 9.01, 2.20 Hz), 6.85 (1H, d, J = 8.64 Hz), 6.71 (1H, dd, J = 7.11, 5.03 Hz), 6.59 (1H, d, J = 5.69 Hz), 3.54 to 3.59 (4H, m), 3.49 (2H, t, J = 6.80 Hz), 2.64 to 2.65 (4H, m), 2.61 (2H, t, J = 7.10 Hz), 2.02 (2H, m). ^13^C NMR δ (ppm) (CH_3_OH-d_4_): 152.5, 149.7, 148.5, 139.3, 136.4, 127.7, 126.0, 124.3, 118.8, 114.8, 109.2, 99.7, 57.5, 54.2, 46.5, 42.6, 26.1. ESIMS [M + H]^+^ calculated for C_21_H_24_ClN_5_ 382.1793, found 382.1784.

### 7-Chloro-*N*-(3-(4-(pyridin-4-yl)piperazin-1-yl)propyl)quinolin-4-amine (compound 22).

The title compound was prepared from compound 5 (0.44 g, 1.40 mmol), 1-(4-pyridyl)piperazine (0.25 g, 1.50 mmol), and triethylamine (0.28 g, 2.80 mmol) according to general procedure A. The crude product was purified by column chromatography on silica, eluting with ethyl acetate-methanol (1:1), to give a tan solid (0.23 g, 43%). HPLC *t*_R_ = 1.72 (99% pure). ^1^H NMR δ (ppm) (CHCl_3_-d): 8.52 (1H, d, J = 5.33 Hz), 8.33 to 8.35 (2H, m), 7.93 (1H, d, J = 2.15 Hz), 7.72 (1H, d, J = 8.93 Hz), 7.21 (1H, dd, J = 8.89, 2.19 Hz), 6.97 (1H, br t, J = 4.40 Hz), 6.68 to 6.73 (2H, m), 6.37 (1H, d, J = 5.39 Hz), 3.39 to 3.47 (6H, m), 2.64 to 2.71 (6H, m), 2.00 (2H, m). ^13^C NMR δ (ppm) (CHCl_3_-d): 24.0, 43.9, 46.1, 53.0, 58.3, 98.7, 108.5, 117.4, 121.6, 124.9, 128.9, 134.8, 149.2, 150.3, 150.5, 152.3, 154.8. ESIMS [M + H]^+^ calculated for C_21_H_24_ClN_5_ 382.1786, found 382.1.

### 7-Chloro-*N*-(3-(4-(pyrimidin-2-yl)piperazin-1-yl)propyl)quinolin-4-amine (compound 23).

The title compound was prepared from compound 5 (0.30 g, 0.95 mmol), 2-(piperazin-1-yl)pyrimidine (0.17 g, 1.05 mmol), and triethylamine (0.19 g, 1.91 mmol) according to general procedure A. The crude product was purified by column chromatography on silica, eluting with ethyl acetate-methanol (1:1), to give a solid (0.16 g, 42%). HPLC *t*_R_ = 6.95 (99% pure). ^1^H NMR δ (ppm) (CH_3_OH-d4): 8.39 (1H, d, J = 5.61 Hz), 8.34 (2H, d, J = 4.76 Hz), 8.11 (1H, d, J = 8.99 Hz), 7.80 (1H, d, J = 2.17 Hz), 7.41 (1H, dd, J = 8.98, 2.18 Hz), 6.62 (1H, t, J = 4.77 Hz), 6.58 (1H, d, J = 5.66 Hz), 3.84 to 3.85 (4H, m), 3.48 (2H, t, J = 6.84 Hz), 2.58 to 2.59 (6H, m), 1.98 to 2.03 (2H, m). ^13^C NMR δ (ppm) (CH_3_OH-d4): 161.5, 157.7, 151.4, 151.1, 148.3, 135.0, 126.2, 124.6, 122.9, 117.4, 109.9, 98.3, 56.1, 52.8, 43.3, 41.1, 24.7. ESIMS [M + H]^+^ calculated for C_20_H_24_ClN_6_ 383.1741, found 383.1745.

### General procedure C for the preparation of compounds 17 and 24 to 27.

Compound 16 was dissolved in acetonitrile and K_2_CO_3_ was added, followed by the appropriate halide. The reaction mixtures were heated to 70°C until thin-layer chromatography (TLC) indicated there was no more compound 16 present. The reaction mixtures were cooled to room temperature and the solvent evaporated. The residue was partitioned between water and chloroform, and the aqueous layer was further extracted with chloroform. The combined organic layers were dried and evaporated to leave a crude product, which was then purified by recrystallization and/or chromatography.

### *N*-(3-(4-Benzylpiperazin-1-yl)propyl)-7-chloroquinolin-4-amine (compound 17).

The title compound was prepared according to general procedure C, from compound 16 (1.2 g, 3.9 mmol), benzylbromide (0.74 g, 4.3 mmol), and K_2_CO_3_ (0.82 g, 5.9 mmol) in acetonitrile (20 ml) and was heated for 16 h. The crude product was purified by column chromatography on alumina, eluting with 100% ethyl acetate, to give a solid (0.4 g, 26%). HPLC *t*_R_ = 7.02 (98%). ^1^H NMR δ (ppm) (CHCl_3_-d): 8.49 (1H, d, J = 5.36 Hz), 7.93 (1H, d, J = 2.15 Hz), 7.82 (1H, d, J = 8.95 Hz), 7.63 (1H, br t, J = 4.02 Hz), 7.35 to 7.36 (5H, m), 7.17 (1H, dd, J = 8.92, 2.18 Hz), 6.30 (1H, d, J = 5.40 Hz), 3.66 (2H, s), 3.36 (2H, td, J = 5.80, 4.09 Hz), 2.62 to 2.64 (10H, m), 1.93 (2H, m). ^13^C NMR δ (ppm) (CHCl_3_-d): 152.2, 150.6, 149.1, 137.3, 134.6, 129.4, 128.6, 128.4, 127.4, 124.6, 122.5, 117.5, 98.4, 63.2, 58.9, 53.7, 52.9, 44.6, 23.3. ESIMS [M + H]^+^ calculated for C_23_H_27_ClN_4_, 395.14970, found 395.19893.

### 7-Chloro-*N*-(3-(4-(5-nitropyridin-2-yl)piperazin-1-yl)propyl)quinolin-4-amine (compound 24).

The title compound was prepared according to general procedure C from compound 16 (1.0 g, 3.3 mmol), 2-chloro-5-nitropyridine (0.55 g, 3.4 mmol), and K_2_CO_3_ (0.54 g, 3.9 mmol) in acetonitrile (15 ml) and was heated for 1.5 h. The crude product was recrystallized from ethanol to give a solid (1.0 g, 71%). HPLC *t*_R_ = 8.05 (98.9% pure). ^1^H NMR δ (ppm) (CHCl_3_-d): 9.07 (1H, d, J = 2.79 Hz), 8.54 (1H, d, J = 5.34 Hz), 8.24 (1H, dd, J = 9.46, 2.79 Hz), 7.95 (1H, d, J = 2.14 Hz), 7.73 (1H, d, J = 8.92 Hz), 7.31 (1H, dd, J = 8.88, 2.16 Hz), 6.78 (1H, br t, J = 4.42 Hz), 6.61 (1H, d, J = 9.49 Hz), 6.39 (1H, d, J = 5.37 Hz), 3.89 (4H, m), 3.44 (2H, td, J = 6.10, 4.47 Hz), 2.67 (6H, m), 2.01 (2H, m). ^13^C NMR δ (ppm) (CHCl_3_-d): 160.3, 152.2, 150.2, 149.1, 146.4, 135.4, 134.8, 133.2, 128.9, 125.0, 121.3, 117.4, 104.6, 98.8, 58.1, 53.2, 44.9, 43.7, 24.1.

### 7-Chloro-*N*-(3-(4-(5-(trifluoromethyl)pyridin-2-yl)piperazin-1-yl)propyl)quinolin-4-amine (compound 25).

The title compound was prepared according to general procedure C from compound 16 (1.0 g, 3.3 mmol), 2-chloro-5-(trifluoromethyl)pyridine (0.63 g, 3.4 mmol), and K_2_CO_3_ (0.54 g, 3.9 mmol) in acetonitrile (15 ml) and was heated for 1.5 h. The crude product was recrystallized from ethanol to give a solid (0.73 g, 56%). ^1^H NMR δ (ppm) (CHCl_3_-d): 8.53 (1H, d, J = 5.35 Hz), 8.35 to 8.52 (1H, m), 7.94 (1H, d, J = 2.15 Hz), 7.76 (1H, d, J = 8.94 Hz), 7.68 (1H, m), 7.25 to 7.29 (~1H, dd, J = 8.98, 2.14 Hz), 7.04 (1H, br t, J = 4.28 Hz), 6.68 (1H, d, J = 9.00 Hz), 6.37 (1H, d, J = 5.39 Hz), 3.76 to 3.77 (4H, m), 3.43 (2H, td, J = 6.01, 4.35 Hz), 2.66 to 2.67 (6H, m), 1.99 to 2.00 (2H, m). ^13^C NMR δ (ppm) (CHCl_3_-d): 160.2, 152.3, 150.3, 149.2, 145.8 (q, JF = 4.19 Hz), 134.71, 134.68 (q, JF = 3.48 Hz), 128.9, 124.9, 124.5 (q, JF = 270 Hz), 121.6, 117.4, 115.7 (q, JF = 32.8 Hz), 105.6, 98.7, 58.4, 53.3, 44.8, 44.0, 23.9. ^19^F NMR δ (ppm) (DMSO-d6): −59.3. ESIMS [M + H]^+^ calculated for C_22_H_23_ClF_3_N_5_, 450.16668; found 450.16557.

### 7-Chloro-*N*-(3-(4-(3,5-dinitropyridin-2-yl)piperazin-1-yl)propyl)quinolin-4-amine (compound 26).

The title compound was prepared according to general procedure C from compound 16 (1.0 g, 3.3 mmol), 2-chloro-3,5-dinitropyridine (0.7 g, 3.4 mmol), and K_2_CO_3_ (0.54 g, 3.9 mmol) in acetonitrile (15 ml) and was heated for 1.5 h. The crude product was recrystallized from ethanol to give a solid (0.5 g, 32%). ^1^H NMR δ (ppm) (CHCl_3_-d): 9.13 (1H, d, J = 2.45 Hz), 8.94 (1H, d, J = 2.45 Hz), 8.54 (1H, d, J = 5.35 Hz), 7.97 (1H, d, J = 2.15 Hz), 7.73 (1H, d, J = 8.93 Hz), 7.40 (1H, s), 6.53 (1H, s), 6.40 (1H, d, J = 5.39 Hz), 3.78 (4H, m), 3.45 (2H, m), 2.70 to 2.65 (6H, m), 2.00 (2H, m). ^13^C NMR δ (ppm) (CHCl_3_-d): 162.2, 161.3, 152.2, 149.8, 146.2, 135.4, 133.8, 132.2, 128.9, 125.5, 121.3, 117.4, 104.6, 98.8, 58.1, 53.2, 44.9, 44.7, 24.2.

### 7-Chloro-*N*-(3-(4-(2,4-dinitrophenyl)piperazin-1-yl)propyl)quinolin-4-amine (compound 27).

The title compound was prepared according to general procedure C from compound 16 (1.0 g, 3.3 mmol), 2,4-dinitrofluorobenzene (0.64 g, 3.4 mmol), and K_2_CO_3_ (0.54 g, 3.9 mmol) in acetonitrile (15 ml) and was heated for 1.5 h. The crude product was recrystallized from ethanol to give a solid (0.94 g, 61%). HPLC *t*_R_ = 8.90 (99%). ^1^H NMR δ (ppm) (CHCl_3_-d): 8.72 (1H, d, J = 2.70 Hz), 8.54 (1H, d, J = 5.33 Hz), 8.30 (1H, dd, J = 9.25, 2.72 Hz), 7.95 (1H, d, J = 2.14 Hz), 7.73 (1H, d, J = 8.91 Hz), 7.34 (1H, dd, J = 8.88, 2.16 Hz), 7.15 (1H, d, J = 9.27 Hz), 6.62 (1 H, br t, J = 4.48 Hz), 6.39 (1H, d, J = 5.37 Hz), 3.43 (2H, td, J = 6.14, 4.54 Hz), 3.36 to 3.39 (4H, m), 2.72 to 2.73 (4H, m), 2.70 (2H, t, J = 5.74 Hz), 2.00 (2H, m). ^13^C NMR δ (ppm) (CHCl_3_-d): 152.2, 150.1, 149.2, 149.2, 138.9, 138.6, 134.8, 129.0, 128.4, 125.0, 123.7, 121.3, 119.6, 117.4, 98.8, 57.8, 52.9, 50.7, 43.5, 24.2. ESIMS [M + H]^+^ calculated for C_22_H_23_ClN_6_O_4_, 471.15313; found 471.15421.

### Inhibition of P. falciparum growth.

CQS (D6) and CQR (Dd2 and 7G8) P. falciparum maintained continuously in culture were used (38). Asynchronous cultures were diluted with uninfected red blood cells (Lampire Biological Laboratories) and complete medium (RPMI 1640 with 0.5% AlbuMAX II) to achieve 0.2% parasitemia and 2% hematocrit. In 96-well microplates, CQ (positive control) or RCQ diluted in complete medium from 10 mM stock in dimethyl sulfoxide (DMSO) was added to the cell mixture to yield triplicate wells with drug concentrations ranging from 0 to 10^−4^ M in a final well volume of 100 μl. After 72 h of incubation under standard culture conditions, plates were harvested and read by the SYBR green I fluorescence-based method (38) using a 96-well fluorescence plate reader (Gemini-EM; Molecular Devices), with excitation and emission wavelengths at 497 and 520 nm, respectively. The fluorescence readings were plotted against log(drug), and the IC_50_s were obtained from curve fitting performed by nonlinear regression using either Prism (GraphPad) or Excel (Microsoft) software. The values obtained for each cell line are normalized to CQ values of 6.9 nM for D6, 102 nM for Dd2, and 106 nM for 7G8.

### *In vitro* heme binding and β-hematin inhibition.

For heme-drug binding studies, a 1 mM stock solution of chloroquine or PL compound was prepared in distilled water, methanol, or DMSO, depending on solubility, and sonicated to ensure complete dissolution. A 5 mM stock solution of heme was prepared by dissolving heme chloride in 0.1 mM NaOH by incubation at 37°C for 30 min. The solution was stored at 4°C for no more than 1 month. At the beginning of each experiment, the stock heme solution was diluted to 5 μM in phosphate buffer (100 mM, pH 5.7) and allowed to equilibrate for 4 h. The 4-h equilibration allowed for the initial heme absorbance to stabilize prior to beginning the titration. Optical titrations with each compound were performed by successive addition of aliquots of its stock solution to the 5 μM heme solution. The pH was monitored throughout the procedure with only negligible (±0.05 pH unit) changes. Equilibrium binding constants were determined by nonlinear least-squares analysis (39).

Hemin chloride (16.3 mg) was dissolved in 1 ml of DMSO. The solution was passed through a 0.2-μm-pore membrane filter to remove insoluble particles and kept at 4°C for no more than 1 month as a stock solution (40). In order to determine the heme concentration of the stock solution, a sample was diluted in 2.5% sodium dodecyl sulfate in 0.1 M NaOH and an absorbance reading was taken at 400 nm. The heme concentration was calculated using Beer's law with a molar absorptivity (ε) of 10^5^ mol liter^−1^ cm^−1^. The optimal heme and Tween 20 concentrations for promoting heme crystallization were calculated by the procedure described by Huy et al. (41). The RCQ compounds were screened for their inhibitory capacity, and IC_50_s were determined. Assays were run in duplicate. A series of solutions were made consisting of 300 μl of various concentrations of the compound under study in 700 μl of 1 M distilled acetate buffer, 300 μl of a 200 μM heme solution freshly buffered by 1 M sodium acetate (pH 4.8), and 200 μl of 0.0375 g/liter Tween 20 solution. This provided a final 40 μM heme solution buffered by 0.67 M sodium acetate at pH 4.8 and 0.0005 g/liter Tween 20, with the test compound ranging in concentration from 0 to 1,000 μM. The mixtures were incubated for 24 h at 37°C (42) and then mixed and transferred to a cuvette for a 415/630-nm absorbance reading. IC_50_s were calculated by (*D*_max_ − *D*_initial_)/2, where *D*_max_ represents the lowest concentration of compound under study to provide maximal absorbance readings, indicating maximal free heme, and *D*_initial_ represents the lowest concentration of drug providing any increase in absorbance over a solution with no drug.

### *In vivo* 3-day suppressive test.

Female CF-1 mice, at 4 to 5 weeks of age, were injected intravenously with 10^6^ erythrocytes infected with P. yoelii (43–45). The following day, and then daily for a total of 3 doses, 5 mice each were administered the appropriate dose of the compound by gavage and evaluated by direct microscopic analysis of Giemsa-stained blood smears (46) 1 day after the final dose. The Portland State University Institutional Animal Care and Use Committee approved the protocols involving animals used in this study.
